# Point-Counterpoint: Cascade reporting—useful tool to support antimicrobial stewardship, or dangerously misleading

**DOI:** 10.1128/jcm.01708-24

**Published:** 2025-06-17

**Authors:** Joseph L. Kuti, Joseph D. Lutgring, Patricia J. Simner, Samia N. Naccache, Emily L. Heil

**Affiliations:** 1 Center for Anti-Infective Research and Development, Hartford Hospital 23893 , Hartford, Connecticut, USA; 2 Division of Healthcare Quality Promotion, Centers for Disease Control and Prevention 549360 , Atlanta, Georgia, USA; 3 Department of Pathology, Division of Medical Microbiology, Johns Hopkins University School of Medicine 1500 , Baltimore, Maryland, USA; 4 Department of Medicine, Division of Infectious Disease, Johns Hopkins University School of Medicine 1500 , Baltimore, Maryland, USA; 5 LabCorp 364534 , Seattle, Washington, USA; 6 Department of Practice, Sciences, and Health-Outcomes Research, University of Maryland Baltimore School of Pharmacy 15513 , Baltimore, Maryland, USA; Vanderbilt University Medical Center, Nashville, Tennessee, USA

## Abstract

Addressing antimicrobial resistance requires multi-disciplinary action, including from the clinical laboratory. Cascade reporting of the antimicrobial susceptibility test (AST) is a strategy used by some laboratories to nudge clinicians toward the use of more narrow-spectrum antimicrobials. Cascade reporting involves suppression of broader-spectrum antimicrobials if the narrower-spectrum first-line antimicrobials show in-vitro susceptibility. Studies have shown that cascade reporting can reduce use of broad-spectrum antimicrobials and reduce antimicrobial resistance rates. However, implementing cascade reporting can be complex, and some question the effectiveness on impacting long-term prescribing behaviors. Furthermore, there are concerns surrounding compliance and the possible negative impact on public health surveillance for antimicrobial resistance. In this article, experts weigh in on the benefits and risks associated with implementing AST cascade reporting. General consensus is that cascade reporting is a benefit to antimicrobial stewardship, but protocols need careful implementation to minimize risk to patients and public health.

## INTRODUCTION

Antimicrobial resistance (AMR) is arguably the largest threat to modern medicine. Recent projections by the Global Burden of Diseases, Injuries, and Risk Factors Study estimated that over 10 million annual deaths globally will be associated with, or directly caused by, AMR infections. Improved care of patients with severe infections, which includes effective infection prevention measures, vaccination, and antibiotic stewardship, could avert 92 million deaths over the next 25 years (1). Strategies for the clinical laboratory to support these efforts include diagnostic stewardship (right test for the right patient at the right time), implementation of rapid diagnostic tests, provider education, generation of cumulative antibiograms, and rational protocols for reporting culture and antimicrobial susceptibility testing (AST) results (2). A widely used, but sometimes controversial, strategy for AST reporting is the use of cascade reporting. The goal behind cascade reporting is to nudge clinicians toward the use of more narrow-spectrum antimicrobials through reporting. The strategy of cascade reporting is to suppress broader-spectrum antimicrobials if the narrower-spectrum, first-line antimicrobials show in-vitro susceptibility (2, 3). Cascade reporting is distinct though complementary to selective reporting, in which antimicrobials are suppressed based on criteria separate from AST results (e.g., organism identification, site of infection, clinical setting, or patient demographic). For example, cefepime, ciprofloxacin, piperacillin–tazobactam, and tobramycin susceptibility might be reported for *Pseudomonas aeruginosa*, while meropenem, ceftolozane–tazobactam, and ceftazidime–avibactam results are suppressed (3). If resistance to cefepime and piperacillin–tazobactam is observed, meropenem susceptibility would be released; if resistance to meropenem is observed, ceftolozane–tazobactam or ceftazidime–avibactam results would be released next. Implementing these cascade approaches can be complex and is often customized by each hospital based on local epidemiology, patient populations, antimicrobial agents on hospital formulary, and priorities of the antimicrobial stewardship team.

In this point–counterpoint, the benefits and risks of implementing AST cascade reporting are debated by experts in this field.

## POINT

### Antimicrobial susceptibility testing and reporting strategies: a simple and valuable antimicrobial stewardship tool

Antimicrobial stewardship aims to optimize clinical outcomes when treating infection while minimizing the unintended consequences of antibiotic utilization, including adverse events, the emergence of antimicrobial resistance, and the selection of new pathogenic organisms (e.g., *Clostridioides difficile*) (4). The testing and reporting of antimicrobial susceptibility testing (AST) results play a paramount role in selecting the optimal drug; therefore, the clinical microbiology laboratory has a responsibility that clearly aligns with antimicrobial stewardship program (ASP) objectives (2, 5, 6). While preauthorization and prospective audit and feedback are recommended and have proven successful, they can be resource intensive (5). Therefore, simple automated interventions that can guide antibiotic selection are also needed. Indeed, the microbiology laboratory’s AST reporting policies within a hospital can influence choice of antibiotic (7). These reporting strategies are built on the concepts of selective and cascade reporting, both of which can be used to improve antibiotic prescribing behaviors. Herein, we provide supportive arguments for why clinical microbiology laboratories in conjunction with other interested parties, such as antimicrobial stewards and infection preventionists, should implement cascade reporting based on facility-specific treatment guidelines.

Cascade reporting is currently recommended by practice guidelines developed by the Infectious Diseases Society of America (IDSA) and the Society for Healthcare Epidemiology of America (5). Evidence that cascade reporting reduces target antibiotic utilization is derived predominantly from quasi-experimental, single-center studies. To reduce unnecessary cefepime days-of-therapy (DOT) for the treatment of ceftriaxone susceptible *Enterobacterales*, Liao and colleagues implemented a ceftriaxone-based cascade reporting algorithm (8). Cefepime results were suppressed unless resistance to ceftriaxone was detected and reported; meropenem results were suppressed unless resistance to both ceftriaxone and cefepime was identified. First-line agents, including ampicillin, ampicillin–sulbactam, cefazolin, ceftriaxone, ciprofloxacin, gentamicin, nitrofurantoin (urine only; an example of a selective reporting strategy), piperacillin–tazobactam, tobramycin, and trimethoprim–sulfamethoxazole, were routinely reported. Over the 1-year study, the investigators observed a 66% reduction in cefepime DOT (1.2 vs 0.8 DOT among patients receiving any antibiotic, *P* < 0.001) after implementation of the cascade reporting algorithm with a small, but statistically significant, increase (1.5 vs 1.7 DOT among patients receiving any antibiotic, *P* = 0.004) in ceftriaxone utilization. No changes in DOT for the other first line reported antibiotics, nor meropenem, which was rarely reported, were observed. Similar results have been reported at other hospitals targeting broad-spectrum antibiotic use against Enterobacterales when compared against a prior control period during which cascade reporting was not in place (9, 10). Additionally, cascade reporting demonstrated effectiveness in improving antibiotic prescribing in older studies (11 – 14), in the treatment of urinary tract, as well as bacteremia and other non-urinary tract infections (15, 16), and, finally, in case-vignette studies (17, 18). In contrast, there are few studies that have not demonstrated effectiveness in antibiotic utilization after implementation of cascade reporting (19).

While the direct goal of cascade reporting is to optimize antimicrobial therapy, additional benefits should not be surprising. Improved susceptibility to the antibiotics targeted for reduced use has been reported across various pathogens (10, 13, 14). Liao and colleagues also reported a statistically significant reduction in hospital length of stay after introducing cascade reporting (14.1 vs 10.9 days, *P* < 0.001), which the authors postulate was due to the ease of transitions of care for patients on ceftriaxone dosed daily compared with broader spectrum agents requiring multiple doses per day (8). While *C. difficile* infection rates have generally not been impacted by cascade reporting (8, 9), it is important to report that no studies to date have demonstrated any adverse effects on clinical response, mortality, reinfection, or resistance rates. In summary, cascade reporting reduces inappropriate use of targeted antibiotics, may improve susceptibility to these agents, and demonstrates no adverse clinical outcomes when implemented properly.

When considering implementation of cascade reporting in the hospital setting, it is paramount that decision-making regarding which agents to cascade, against which pathogen, and when (i.e., in what order to release results) be a multi-disciplinary process that includes interested parties at the institution. These would include members of the clinical microbiology laboratory, antimicrobial stewardship program, pharmacy department, infectious diseases division, as well as other specialty practitioners that may have direct influence on antibiotic prescribing behaviors, as needed. For example, ophthalmology may be involved when considering selective and cascade reporting strategies for infections affecting the eye due to limited topical agents available for management. Additionally, the information technology department or those individuals responsible for coding and manipulation of the laboratory information system (LIS) and reporting into the electronic medical record (EMR) should be part of the team. Historically, guidance on susceptibility testing and reporting has been limited (20). More recently, the Clinical and Laboratory Standards Institute (CLSI) has updated its CLSI M100 in an attempt to provide the laboratory initial guidance on testing and reporting of specific antimicrobials against specific bacteria (3, 21). Table 1 has reclassified individual antimicrobials into Tiers instead of Groups, with each Tier representing an opportunity for laboratories to routinely test (or by request only) and when to report, either routinely, selectively, or by cascade reporting. Once again, these examples are guidance to the laboratory, and a multidisciplinary team should be engaged in identifying when and how cascade reporting would provide the greatest value for the institution. The guidelines provided in Table 1 should be considered at each institution when devising reporting strategies that reflect the antimicrobials on formulary, local treatment guidelines, antimicrobial resistance rates, and ASP resources available to the facility (21). As another example, the Royal College of Pathologists of Australia has developed selective reporting guidelines for laboratories in Australia and New Zealand, and these may serve as a template for others looking to implement selective and cascade reporting at their institution (22). Finally, as resources may vary for ASP depending on the type (e.g., community, academic, etc.) or size of hospital, it should be highlighted that AST reporting strategies are simple, effective, and affordable strategies that can be applied by any-size hospital or laboratory to complement an ASP as it is not resource intensive.

Despite the simplicity and effectiveness of cascade reporting, some caveats should be initially considered during implementation. First, the ability for the LIS or EMR to automatically follow the intended algorithm without any required manual manipulation is critical. If manual manipulation is required, the time and resource commitment may be too burdensome. As an example, when developing the annual antibiogram, if specific antibiotic results are only available if they were reported by the LIS/EMR, this could provide misleading resistance rates for those agents that are least reported since, presumably, their susceptibility results would be only tabulated against the most resistant isolates at the institution. Ensuring that the laboratory and ASP have access to all results, instead of just those reported, can save significant time at the end of the year when creating the antibiogram. Strategies to account for cascade reporting rules when assembling the antibiogram are provided in the CLSI M39 document on *Analysis and Presentation of Cumulative Antimicrobial Susceptibility Test Data* (23). In addition to affecting local antibiograms, cascade reporting has been demonstrated to bias national surveillance data (24). In data submitted to the National Healthcare Safety Network Antimicrobial Resistance Option, there is evidence of selective and cascade reporting, and facilities appearing to report results in a cascade manner appear to have lower rates of susceptibility to some suppressed drugs compared to facilities reporting all results. As an example, for facilities with such apparent selective reporting, urinary *Escherichia coli* isolates were 62.7% susceptible to cefepime, while for non-selective reporting facilities, urinary *E. coli* isolates were 92.8% susceptible to cefepime (24). There are potential solutions, however, and results can be inferred for surveillance purposes as has been done previously (25).

Next, common antibiotics that are cascaded but are confirmed resistant should have their results released (e.g., cefepime susceptible, meropenem-resistant *P. aeruginosa*). This would avoid the empiric assumption of a susceptible result for what might be considered more broad-spectrum agents when alternative resistance mechanisms are recognized that may cause these discrepancies or when unusual/unexpected resistant results are confirmed. Finally, cascaded antibiotics will likely be viewed less commonly; therefore, their absence from reports may create an education gap as to the spectrum and utility of these last-line agents. While most of these antibiotics may be restricted to an ASP or infectious diseases consultation, well-designed educational tools for the front-line prescribers should be developed to interpret results properly.

In summary, the clinical microbiology laboratory can implement cascade and selective reporting strategies in association with the antimicrobial stewardship program to significantly impact antibiotic prescribing behaviors. Few stewardship strategies are as feasible and effective as directed antibiotic utilization using currently available LIS/EMR technology. As a result, cascade and selective reporting should be evaluated, customized to the institution’s needs, and operationalized by clinical microbiology laboratories in conjunction with interested partners to aid in improving the treatment of infections in hospitalized patients.

**Joseph L. Kuti, Joseph D. Lutgring, Patricia J. Simner**

## COUNTERPOINT

### Cascade reporting con: you CAN handle the truth

As outlined in the pro side of this debate, collaboration between the clinical microbiology laboratory and antimicrobial stewardship has a positive impact on antimicrobial prescribing patterns. While generally supportive of targeted cascade reporting, there are concerns associated with sustainable implementation. We present considerations from the perspective of the clinician, the patient, and the laboratory, which we propose are essential to tackle before institutional implementation of cascade reporting.

Cascade reporting is a strategy of passive “nudging” to guide clinician behavior. While this type of intervention may achieve the desired outcome, in this case utilizing narrow-spectrum antimicrobials when organisms are susceptible to these antimicrobials *in-vitro*, it is likely to go unnoticed by the clinician, which does not improve or change behavior long term and does raise ethical concerns (26). There is a lack of transparency inherent in suppressing otherwise correct susceptibility reporting, as the clinician does not know that there are certain results not being shown to them. The lack of transparency can be problematic for several reasons. First, if the clinician is unaware of the intervention, it is unlikely to instill actual education and behavioral changes in prescribing practices. If the cascade reporting strategy was ‘turned off’ (e.g., for the purpose of being updated), or the clinician was at a site that did not use the strategy (e.g., interpreting results from an outpatient clinic or separate encounter), prescribing behaviors would likely return to baseline. Instead of simply hiding results from clinicians, building education into the culture reports could result in more sustainable practice changes.

Second, suppressing some results without transparency might lead to false assumptions regarding susceptibility patterns of the non-reported antimicrobials. For example, if anti-pseudomonal beta-lactams are suppressed for Enterobacterales that are susceptible to third-generation cephalosporins, a clinician might assume that a ceftriaxone-susceptible *E. coli* is susceptible to piperacillin–tazobactam when in fact it harbors an inhibitor-resistant TEM beta-lactamase and is piperacillin–tazobactam resistant (27). Other examples include suppressing daptomycin in vancomycin-susceptible *Enterococcus* isolates or suppressing gentamicin and only reporting tobramycin where assumptions about susceptibility are made. Without having the ‘full picture,’ it can be difficult to phenotypically guess resistance mechanisms in gram-negative organisms. Providers might also make incorrect assumptions about the susceptibility of antimicrobials and antimicrobial classes that have not been tested at all by the lab. Additionally, there are circumstances when patient factors (e.g., allergies) may dictate use of a less-favorable suppressed agent. Finally, in the case of polymicrobial infections where organisms of varying resistance profiles are co-isolated within one sample, clinicians want to be assured that the appropriate antimicrobial selected for the more resistant organism has been tested and is susceptible for the less resistant organism. It is common practice for clinicians to call the lab to request the release of suppressed antimicrobials, which takes an unacknowledged toll on the laboratory, especially in the current era of severe laboratory short staffing (28).

The impact of cascade reporting on the completeness of the patient’s electronic medical record (EMR) must also be considered. Patients are followed longitudinally and often progress through different institutions whether within the same system, from inpatient to outpatient settings, or between different institutions served by the same or different laboratories. Unless the full susceptibility panel results are available for patients and their providers, if cascade rules result in truncated reports into the EMR, the clinical microbiology laboratory is limiting tested information. The 21st Century Cures Act requires that all results tested be released to patients, and that an individual has the right to request and receive a copy of their protected health information (29). Exceptions to this rule include the “Preventing Harm Exception” (45 CFR Part 171). Prior to implementing cascade rules, the regulatory framework for allowing suppression may need to be established by each institution to include whether the suppression of these rules falls under the “Preventing Harm Exception” clause of the Cures act.

On an institutional level, clinical microbiology laboratories are mandated to provide accurate antibiograms with stakeholders from infection prevention and the extended antimicrobial stewardship team. Any algorithm for antimicrobial suppression needs to preserve the ability of the laboratory to accurately report the percentage of organisms that are susceptible to antimicrobials tested. Suppression associated with cascade reporting can occur informatically at one of 4 levels: AST Instrument, Laboratory Information System (LIS), EMR, EMR middleware/third-party software. Antibiograms can be unaffected by cascade reporting if the suppression occurs informatically downstream of where antibiogram data are collected but would be incomplete if antibiogram data are generated from the results reported to providers when certain data are suppressed. In addition, institutions are obligated to submit antimicrobial resistance surveillance information to public health entities, such as the Center for Disease Control and Prevention’s National Healthcare Safety Network database, and, if cascade reporting is utilized upstream of these data, this can weaken public health efforts to gather representative antimicrobial resistance data and create inaccurate cumulative antibiograms (24).

Many healthcare institutions are supported by reference laboratories instead of onsite affiliated clinical microbiology resources. Reference laboratories have an obligation to report all antimicrobials tested on their panels if they are appropriate for the originating source and the specific organism and would risk running afoul of the Cures act if viable results are actively withheld. Reference labs generally create a charge for the AST panel tested and not individual drugs, and panel utilized counts as a single procedure associated with a single CPT code, regardless of how many drugs are released. However, releasing only specific drugs depending on the susceptibility picture would result in a challenging reporting structure for both the lab and client institution. The client institution could request the release of previously suppressed results after the fact, but this request will generally require a separate authorization, even if it does not engender a separate charge, and would be subject to the availability of the original data. Reference labs also serve different institutions with different patient populations, regulatory requirements, formularies, and stewardship priorities. Establishing different suppression rules for different client institutions would increase the complexity of the cascade and with it the risk of patient harm and require significant investment in additional computational solutions.

There are underappreciated complexities with the successful implementation of cascade reporting. First, one ideally would have separate access for the institution’s stewardship, infection prevention, and infectious diseases teams to view the full panel of susceptibilities. This is important for epidemiologic/surveillance reasons and to allow expert access to the full panel of information to help with decision making for scenarios that do not fit the cascade rules. Second, the rules need to be customized by specimen type (e.g., blood vs urine), pathogen type, and other factors, such as patient age. That complexity can make it difficult to make changes once the rules are in place—both room for error but also inertia if the rules need to be relaxed or changed. Updating one suppression rule can inadvertently trigger issues elsewhere. Additionally, for hospitals working as systems, some hospitals within the system may use different laboratories and have different AST platforms, making the creation of uniform rules challenging. A champion responsible for reviewing these rules regularly for appropriateness is needed.

Potential solutions that allow for the safeguarding of the laboratory’s processes while maintaining the benefits of cascade reporting need to be developed. A solution that exists in some EMRs is for the laboratory to release all routinely tested appropriate organism/antimicrobial combinations for source/organism into the LIS but for the institution to rely on suppression programming within the EMR to implement cascade rules. This allows for all results to be visible to providers with specific credentials (e.g., infectious diseases clinicians) but does require significant information technology investment and potential use of middleware (e.g., EPIC’s Bugsy, previously known as EPIC Infection Control [30]). This strategy preserves complete data for both antibiograms and reporting to public health entities while still allowing institution-specific rules for forward-facing data sharing. Other innovative solutions, which would allow clinicians to internalize antimicrobial stewardship lessons, would be for antimicrobials to be reported by class and then order of recommended use (i.e., from narrow to broad). Alternatively, antimicrobials slated for cascade reporting suppression could be “grayed out,” or first-line antimicrobials could be reported in bold. While these changes require IT investment in implementation into the EMR, the microbiology community is capable of tackling and advocating for this change given the documented beneficial impacts of cascade reporting on antimicrobial use (8).

There are several unmet research needs with cascade reporting that should be addressed. The majority of studies focus on the impact of cascade reporting on antimicrobial prescribing inpatient settings, with fewer studies evaluating ambulatory or outpatient settings, impact on unintended consequences, or microbiologic epidemiology (31, 32). One systematic review of 20 studies evaluating cascade reporting found that only four studies evaluated appropriateness of antimicrobial prescriptions or potential adverse outcomes related to cascade reporting implementation (31). From these studies, minimal or no impact was seen on important clinical outcomes, such as *C. difficile* infection rates or antimicrobial resistance, which would be more impactful outcomes beyond just select antimicrobial consumption alone.

In addition, the majority of the data for the effectiveness of cascade reporting comes from large academic medical centers that presumably have an on-site microbiology laboratory, a clinical microbiology director, and a dedicated antimicrobial stewardship team that manages the majority of therapeutic decisions, has a full stake in the cascade reporting algorithm chosen, and a full understanding of the available antimicrobial susceptibility panels available to the institution. Success is more difficult to extrapolate to institutions with limited antimicrobial stewardship support, which limits generalizability across all practice settings.

How do we make cascade reporting work for all? We encourage laboratories to report all appropriate AST results and work toward suppression within the EMR, which can be achieved by increasingly available EMR middleware. When possible, we encourage transparency regarding antimicrobials that are suppressed, including posting suppression rules in visible locations within the EMR. We hope that the increased visibility on the importance of cascade reporting allows for the creation of innovative reporting functions within the EMR.

**Samia N. Naccache and Emily L. Heil**

## SUMMARY

The authors achieve consensus that AST reporting strategies do impact antimicrobial utilization, with no evidence that this practice may have adverse effects on patient outcomes, such as clinical cure, mortality, or antimicrobial resistance rates. The authors also agree that consensus-driven policies developed by the laboratory in conjunction with ASP, infectious diseases, and other key parties are key to the successful implementation of cascade reporting strategies. Recent publication of guidance from the Clinical and Laboratory Standards Institute can serve as a launching point for these discussions by providing a starting point of cascade protocols. The authors also agree that implementation of cascade reporting can be challenging, requiring sometimes complicated information technology solutions to enable the automated reporting algorithms central to cascade reporting strategies. The authors also point out the potential pitfalls of cascade reporting on skewing antibiogram data generation if antibiograms are derived from reported AST results rather than from all tested AST results. Similarly, depending on how cascading is implemented (hospital information system or laboratory AST system middleware), AMR rates reported to national surveillance programs may be skewed by the cascade results. These challenges, however, are surmountable with sufficient energy, resources, and dedication. Indeed, many institutions have successfully implemented cascade reporting.

More complex to resolve is the debate over whether cascade reporting actually supports one of the key missions of an effective antimicrobial stewardship program: education. On the one hand, cascade reporting is an aid to decision-making for increasingly busy clinicians. On the other hand, cascade reporting is a crutch, preventing clinicians from developing more sophisticated decision-making skills when it comes to appropriate antimicrobial prescribing. While nudging strategies, like cascade reporting, have been consistently shown to effectively change behavior toward a desired endpoint (i.e., prescription of narrow-spectrum antimicrobials), there is concern that this form of behavior modification is non-educative. Because the clinician is unaware of the nudge, they do not incorporate the desired practice into future decision-making (33), which may minimize the overall impact of the intervention for scenarios where a cascade report is not available. Education and nudging do not have to be mutually exclusive. If incorporated thoughtfully with appropriate education, it may be possible to achieve endpoints of both immediate effect (response to the AST report) and sustained behavior (future similar prescribing practice).

It is clear that AMR is a global threat that disproportionately affects countries outside North America and Europe (1). Tools for the implementation of rational AST reporting provided by IDSA, CLSI, and others are not applicable to many countries most affected by AMR due to limitations of antimicrobial availability or access to laboratory testing and AST as a whole. It is also clear that without intense intervention, a key component of which is antimicrobial stewardship, AMR will threaten most, if not all, medical advancements made in the past century (2). Laboratories can and must play a leading role in the preservation of antimicrobials through evidence-based practices surrounding how laboratory tests are used and how the results are reported. Cascade reporting may form an important piece of this strategy, which, without doubt, must occur in the context of close collaboration across the multi-disciplinary team responsible for antimicrobial stewardship.

**Romney M. Humphries, Point-Counterpoint Editor,** ***Journal of Clinical Microbiology***
